# SARS-CoV-2 Seroprevalence of Surinamese Children and Determinants of Seropositivity in the CCREOH/MeKiTamara Cohort

**DOI:** 10.3390/children13040493

**Published:** 2026-03-31

**Authors:** Delmaliz Barreto-Vázquez, Jeanine M. Buchanich, Ernesto T. A. Marques, Hannah H. Covert, Firoz Abdoel Wahid, Ashna D. Hindori-Mohangoo, Wilco C. W. R. Zijlmans, Arti Shankar, Maureen Y. Lichtveld

**Affiliations:** 1 Department of Environmental and Occupational Health, School of Public Health, University of Pittsburgh, Pittsburgh, PA 15261, USA; fza3@pitt.edu; 2 Department of Biostatistics and Health Data Science, School of Public Health, University of Pittsburgh, Pittsburgh, PA 15261, USA; jeanine@pitt.edu; 3 Department of Infectious Diseases and Microbiology, School of Public Health, University of Pittsburgh, Pittsburgh, PA 15261, USA; marques@pitt.edu; 4 Department of Virology and Experimental Therapeutics, Aggeu Magalhães Institute, Oswaldo Cruz Foundation, Recife 50740465, Brazil; 5 Independent Researcher, Swarthmore, PA 19081, USA; hcovert@hotmail.com; 6 Foundation for Perinatal Interventions and Research in Suriname (Perisur), Anton Dragtenweg 93, Paramaribo, Suriname; ashna.mohangoo@perisur.org; 7 Department of Pediatrics, Faculty of Medical Sciences, Anton de Kom University of Suriname, Prof. W.J. Flustraat 5, Paramaribo P.O. Box 537, Suriname; wilco.zijlmans@uvs.edu; 8 Department of Biostatistics and Data Science, Tulane University School of Public Health and Tropical Medicine, New Orleans, LA 70112, USA; sarti@tulane.edu

**Keywords:** Suriname, CCREOH cohort, COVID-19, SARS-CoV-2, seroprevalence, children, social determinants of health

## Abstract

**Highlights:**

**What are the main findings?**

- We found an increase in SARS-CoV-2 antibody prevalence among children within the CCREOH/MeKiTamara between 2021–2022 and 2023.
- Children from unvaccinated mothers were more likely to be SARS-CoV-2 seropositive by 2023.

**What is the implication of the main finding?**

- Identifying the factors associated with prior COVID-19 infection among young Surinamese children will better inform local pediatric interventions and vaccine policies.
- The SARS-CoV-2 transmission among Surinamese children was explosive yet silent over the 22 month average interval between 2021 to 2023.

**Abstract:**

Background/Objectives: The main goal of this study is to identify predictors associated with Severe Acute Respiratory Syndrome Coronavirus 2 (SARS-CoV-2) seropositivity in children, including demographics, history of coronavirus disease 2019 (COVID-19) infection of the child and the household members, prevention practices, and maternal vaccination. Methods: This retrospective cross-sectional study within the Caribbean Consortium for Research in Environmental and Occupational Health (CCREOH)/MeKiTamara cohort included 300 mother-child dyads recruited in Paramaribo and Nickerie, Suriname (February–April 2023). The total immunoglobulin G (IgG) anti-spike domain 1 (S1) and anti-nucleoprotein (NP) were quantified in dried blood spot (DBS) eluates from children using indirect enzyme-linked immunosorbent assays (ELISAs). Demographic information, COVID-19 prevention measures, history of viral infection of the child and the household members, and COVID-19 vaccination questionnaire data were recorded. Predictors of SARS-CoV-2 seroprevalence were determined using binary logistic regression. Results: Among 278 seropositive children in 2023, 73.4% were in the 5–6-year-old age group, 54.7% were female, 36.3% were of Asian descent, and 69.8% were recruited in Paramaribo. Seroprevalence increased from 33.8% in 2021–2022 to 93.3% in 2023, with a mean follow-up of 21.5 months. Of the 100 children previously tested by Polymerase Chain Reaction (PCR) or antigen test, 25 had confirmed COVID-19, as reported by mothers. Children from unvaccinated mothers were 6.11 times more likely to be seropositive (*p* = 0.022). Conclusions: This study shows a significant increase in SARS-CoV-2 seropositivity in Surinamese children aged 3–6 years between collection periods, indicating multiple exposures. Future public health interventions and policies should account for maternal vaccination status to reduce children’s exposure to COVID-19 during future outbreaks.

## 1. Introduction

During the COVID-19 pandemic, pediatric cases accounted for a small proportion of cases worldwide [1]. However, in low-resource regions, many cases were likely undiagnosed and unreported [2]. A systematic review and meta-analysis study identifying SARS-CoV-2 seroprevalence and its determinants in children globally found that low- and middle-income countries (LMICs) have the highest pooled seroprevalence estimates (21.21%) compared to high-income countries (10.02%) across six pandemic waves (ranging from late 2019 to April 2022) [3]. In Latin America and the Caribbean, 3.56% of children were seropositive in the first wave (late 2019 to June 2020) and 21.87% in the fourth wave (April 2021 to June 2021) of the pandemic [3]. In addition, the Delta and Omicron variant periods were characterized by high viral transmission [4,5,6], which facilitated the increase in pediatric cases and hospitalizations [3,7,8,9].

Community-based studies reported associations between a high SARS-CoV-2 seropositivity in children and a previous history of infection [2,7], household exposure and composition [7,10,11], or socioeconomic and demographic background [2,7]. A cross-sectional study in Amazonian Brazil reported that children from poor households and less educated mothers were significantly more likely to be seropositive at the age of 5 years [2]. In addition, the prevalence of COVID-19 increased among children experiencing food insecurity and those born to non-White mothers [2]. SARS-CoV-2 transmission from household members and caregivers was associated with an increased likelihood of seropositivity in children from Brazil [2] and India [7]. Large, overcrowded households in urban areas were a major risk factor for SARS-CoV-2 seropositivity in children from Mérida, Mexico [11], and Lima, Peru [10].

Pediatric data from Suriname, in northern South America, remain sparse. Limitations in testing, disease surveillance, and vaccine coverage have hindered the complete assessment of SARS-CoV-2 impacts on Surinamese children and their social and behavioral determinants [12,13]. To address these gaps, the current study aims to determine the SARS-CoV-2 seroprevalence from natural infection of children aged 3 to 6 years old enrolled in the CCREOH/MeKiTamara [14] cohort at collection time point 1 (C1) from 3 January 2021 to 19 September 2022, and collection time point 2 (C2) from 21 February 2023 to 22 April 2023, and identify behavioral and social determinants of SARS-CoV-2 seropositivity in children in 2023. We hypothesize an increase in SARS-CoV-2 seroprevalence between the two time periods. We also hypothesize that a history of COVID-19 infection, compliance with child-specific social distancing practices, and maternal COVID-19 vaccination are significant factors associated with SARS-CoV-2 seropositivity of children.

## 2. Materials and Methods

### 2.1. Study Design and Participants

**Figure 1:**
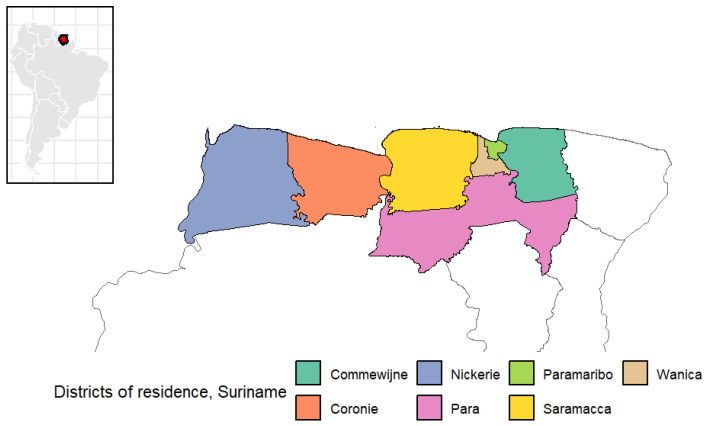
Districts of residence of participants in the study.

This retrospective cross-sectional study within the CCREOH/MeKiTamara cohort collected data from 300 mother-child dyads recruited in Paramaribo and Nickerie, Suriname, between February and April 2023. The CCREOH prospective environmental epidemiologic cohort study investigates the effects of chemical and non-chemical environmental exposures on mother-child dyads in Suriname [14]. This study used existing dried blood spot (DBS) samples collected in 2021–2022 by the CCREOH study. Inclusion criteria for this study were mother-child dyads enrolled in the CCREOH/MeKiTamara cohort and living in the following districts of Suriname: Paramaribo, Para, Wanica, Commewijne, Saramacca, Nickerie, and Coronie (Figure 1). Exclusion criteria included individuals who did not wish to participate in the current study, those who had moved to another country, and those not currently residing in the districts mentioned above. Participants were randomly selected from the CCREOH/MeKiTamara prospective cohort based on a sample size calculation of n = 281 for a logistic regression model with a small effect size (odds ratio (OR) = 1.53) for 80% power at the 5% level of significance for a two-tailed test. To account for the loss of participants due to exclusion criteria, 337 mothers were called and invited to participate, of which 300 were recruited. Participants living in Paramaribo (N = 120), Para (N = 11), Wanica (N = 68), Commewijne (N = 8), and Saramacca (N = 2) districts were recruited at the Diakonessenhuis hospital in Paramaribo. Participants from Nickerie (N = 90) and Coronie (N = 1) were recruited at Zuster D. Dankers Centrum, Nickerie. COVID-19 vaccination for children under 12 years was not authorized in Suriname during the study period.

### 2.2. Questionnaires

Interviewer-assisted questionnaires were administered by four trained research assistants and community health workers between 21 February 2023 and 22 April 2023 (this collection time range was defined as C2). Responses were recorded in the web platform Research Electronic Data Capture (REDCap) version 12.2.11 (University of Pittsburgh Clinical and Translational Science Institute (CTSI) Grant Number UL1-TR-001857). The questionnaire (See Supplementary Materials) included items about demographic information, COVID-19 prevention measures, history of COVID-19 infection of the child and the household members, and COVID-19 vaccination data adapted from previous studies [15,16,17,18,19,20]. The items were culturally tailored to the Surinamese context, translated into Dutch, and pre-tested by the local and US-based study team.

### 2.3. DBS Processing

Children’s blood drops were collected on Whatman™ 903 cards (Fisher Scientific, Waltham, MA, USA) by trained research assistants and stored at −20 °C until shipped to the US for analysis. This study used existing DBS collected in C1 by the CCREOH study, which were stored and shipped in a similar manner. Twenty 3 mm discs were punched from two circles of each card using a pneumatic puncher (Analytical Sales and Services Inc., Flanders, NJ, USA). Discs were incubated shaking in 400 µL of dilution buffer (phosphate-buffered saline (PBS) 1X containing 0.1% (*v*/*v*) Tween 20 and 0.1% (*w*/*v*) Bovine Serum Albumin (BSA)) at 4 °C for 24 h, centrifuged two times, and eluted to approximately 250–260 µL. DBS eluates were then stored at −80 °C.

### 2.4. SARS-CoV-2 Serology

Total IgG anti-SARS-CoV-2 domain 1 (S1) and nucleoprotein (NP) were detected by in-house ELISAs [21]. A total of 50 µL of recombinant NP of SARS-CoV-2 (RayBiotech, cat# 230–30164–100, Peachtree Corners, GA, USA) or recombinant S1 (Sino Biological, cat# 40591-V08H, Houston, TX, USA) at 2 µg/mL in carbonate/bicarbonate buffer was used to coat 96-well plates and incubated in a humid chamber at 4 °C overnight. The next day, plates were aspirated and then blocked with blocking buffer (PBS 1X containing 0.1% (*v*/*v*) Tween 20 and 5% (*w*/*v*) BSA) for 1 h at room temperature. We used pre-pandemic pooled samples from healthy individuals as negative controls and PCR-positive samples from hospitalized COVID-19 patients as positive controls for the assays, diluted at 1:100 in dilution buffer [21]. DBS eluate samples were diluted to 1:10 in dilution buffer. Controls and samples were transferred in duplicates of 50 µL for each well and incubated at room temperature for 1 h. The plates were washed 6 times. The secondary antibody (Goat Fab2 anti-human IgG (H + L)-HRP; Jackson Immunoresearch, cat# 109–036–003, West Grove, PA, USA) was diluted 1:20,000 in dilution buffer, then added and incubated at room temperature for 1 h. Plates were washed, soaked for 5 min in washing buffer (PBS 1X with 0.1% (*v*/*v*) Tween 20), and aspirated. Fifty µL of KPL SureBlue Reserve™ TMB Microwell Peroxidase Substrate (SeraCare; cat# 53–00–03, Gaithersburg, MD, USA) were added to the wells and incubated for 20 min, then we added 50 µL of 1 N HCl per well was added to stop the reaction. Plates were screened for total IgG anti-SARS-CoV-2 NP and S1 by measuring absorbance at an optical density (OD) of 450 nm in the microplate reader. ELISA results were validated by calculating a coefficient of variation below 20% for the replicates on each plate. ODs were adjusted by subtracting the blank value. To analyze total IgG levels, first, we first calculated the cut-off for each plate. The Sample/Cut-off (S/C) ratio was then calculated.

Cut-off = Adjusted Average OD of the Negative Control + 3x(STDEV Negative Control) (1)

S/C ratio = Sample-adjusted OD450 nm/cut-off for the plate (2)

Samples with a ratio >1 were identified as seropositive and ≤1 as seronegative.

### 2.5. Seroprevalence Analyses

**Figure 2:**
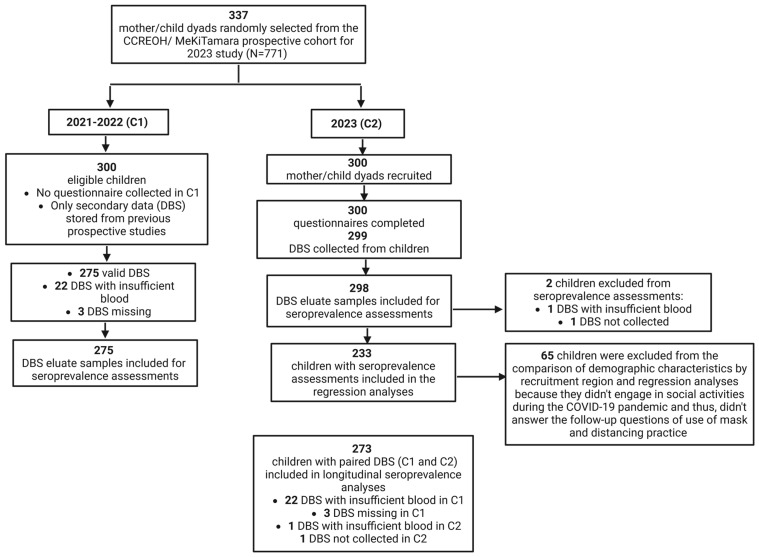
Recruitment of participants and data collection. C1, collection time point 1 from 3 January 2021 to 19 September 2022; C2, collection time point 2 from 21 February 2023 to 22 April 2023; N, sample size; CCREOH, Caribbean Consortium for Research in Environmental and Occupational Health; DBS, dried blood spots.

A flowchart of the serological analyses is shown in Figure 2. A total of 273 paired samples from C1 and C2 were analyzed for longitudinal seroprevalence of NP and S1 antibodies. Children who retained seropositive status were SARS-CoV-2 seropositive in both C1 and C2. Those who seroconverted were seronegative in C1 and seropositive in C2. Those who seroreverted were seropositive in C1 and seronegative in C2. Children who retained seronegative status were SARS-CoV-2 seronegative in both C1 and C2.

### 2.6. Statistical Analysis

Continuous variables were described as means and SDs, and categorical variables as frequency counts and/or percentages. Due to lower enrollment in some of the districts of residence of participants, we aggregated the districts by recruitment region: Paramaribo and Nickerie. Other aggregated variables are listed in Table A1. Comparison of demographic variables by recruitment region was assessed using Mann–Whitney U tests or Pearson’s Chi-squared test (χ^2^). Mann–Whitney U tests were used to cross-sectionally compare the S/C ratios of total IgG anti-NP or S1 at C1 (N = 275) vs. C2 (N = 298) and the antibody S/C ratios of children nose swab tested for COVID-19 at any point in time until C2 (nose swab negative N = 75; nose swab positive N = 25). Wilcoxon matched pairs tests were used to longitudinally compare the S/C ratios of total IgG anti-NP or S1 between paired samples from C1 to C2 (N = 273 pairs). Spearman analysis was used to identify correlations between total IgG anti-NP and/or S1 S/C ratios at C1 and C2, and between total IgG anti-S1 S/C ratio and age of child at C2. We used Wilson’s score with 95% confidence intervals (CI) to estimate the crude seroprevalence of IgG anti-S1, stratified by age of children, gender, and recruitment region. Cohen’s kappa test assessed the concordance between ELISA serology results of total IgG anti-NP and anti-S1 of C1 and C2 and evaluated sensitivity and specificity.

We assessed the association between total IgG anti-S1 seroprevalence of C2 (outcome) and each of the 14 predictors (i.e., behavioral and social determinants) using univariate regression. Before building the binary regression models, we assessed interaction effects. Models with interactions were compared with models containing only main effects using the likelihood ratio test (LRT; Table A2). A *p* < 0.05 indicated that the interaction was necessary for the model. The predictors were included in the binary logistic regression based on the following criteria: univariate analysis with *p* ≤ 0.2 (age of mother and household size) plus those predictors hypothesized to be associated with SARS-CoV-2 seroprevalence (COVID-19 diagnosis by healthcare provider, social activities of children during the COVID-19 pandemic, use of mask by child, distancing practices by child, and mother COVID-19 vaccination). Two binary logistic regression models were compared using the LRT; one with all the variables following the criteria mentioned above, but without interaction, and another including the interaction. An LRT with *p* < 0.05 was considered for the inclusion of the interaction in the final model. However, if the interaction compromised the model stability or validity, then it was not included in the final model. The final model was validated by assessing its accuracy, sensitivity, and specificity, Area Under the Curve (AUC), and Receiver Operating Characteristic (ROC) curve, goodness of fit, and Variance inflation factor (VIF). Adjusted odds ratios were calculated for the coefficients included in the final model.

Correlations between variables included in the final model were assessed using the Pearson’s Chi-squared test (χ^2^), with a significance of *p* < 0.05. The correlations tested were the following: maternal COVID-19 vaccination status and COVID-19 positive household contacts, distancing practices by the child and maternal COVID-19 vaccination status, COVID-19 diagnosis of the child by a healthcare provider and maternal COVID-19 vaccination status, household size and maternal COVID-19 vaccination status, household size and COVID-19 positive household contacts, household size and COVID-19 diagnosis of the child by a healthcare provider, and COVID-19 diagnosis of the child by a healthcare provider and COVID-19 positive household contacts. Initial variable recoding (transformation) was performed in IBM SPSS version 29. Statistical analysis for serology was performed in GraphPad Prism version 10.0.3. All other statistical analyses were performed in R version 4.4.2.

## 3. Results

### 3.1. Demographic Comparisons of Study Participants

**Figure A1:**
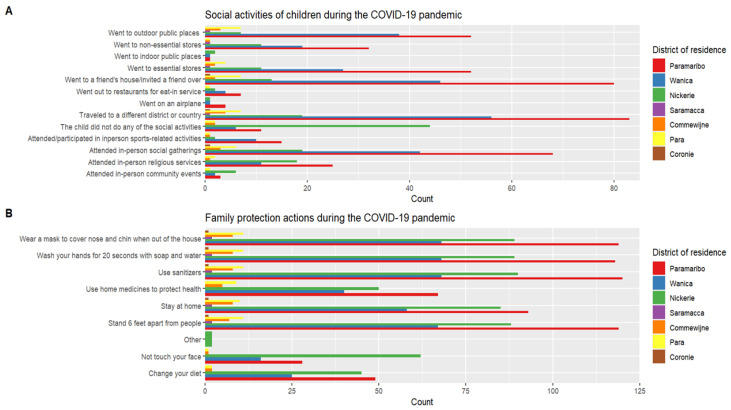
Social activities of children and family protection actions by district of residence (N = 300). (**A**) Social activities of children during the COVID-19 pandemic by district of residence reported in C2. (**B**) Family protection actions during the COVID-19 pandemic by district of residence reported in C2. Participants from Paramaribo (N = 120), Para (N = 11), Wanica (N = 68), Commewijne (N = 8), and Saramacca (N = 2), Nickerie (N = 90), and Coronie (N = 1).

Table 1 shows the comparison of participants’ characteristics by recruitment region at C2. Overall, the mean age of children was 4.8 years (standard deviation, SD 0.5). About 132 children were female, 67 of African descent, and 130 were living in large households of 5 or more members. Overall, the mean age of mothers was 33.7 years (SD 6.4); 124 had none/primary/lower secondary education; 168 were employed; and 134 were COVID-19 vaccinated. About 118 children engaged in 3 or fewer social activities during the COVID-19 pandemic. The specific social activities of children and family protection actions by the district of residence are shown in Figure A1. For children who engaged in social activities, 162 reported using masks ‘Often/Always’, while 124 reported having ‘Never/Rarely’ practiced distancing. About 166 were not diagnosed with COVID-19 disease by a healthcare provider, and 118 reported COVID-19-positive household contacts. About 161 participants reported practicing 6 or more family protection actions during the COVID-19 pandemic.

Children recruited in Paramaribo were, on average, slightly older than those recruited in Nickerie (mean 4.8 years [SD 0.4] vs. 4.5 years [SD 0.7], respectively; *p* < 0.001). Ethnicity varied by region, with a higher proportion of African descent participants recruited in Paramaribo compared to Nickerie (44.7% vs. 6.4%, respectively; *p* < 0.001). A higher proportion of children engaging in four or more social activities during the COVID-19 pandemic were recruited in the Paramaribo region compared to Nickerie (56.4% vs. 23.4%, respectively; *p* < 0.001). Distancing was less frequently practiced by children recruited in the Paramaribo region compared to those in Nickerie (Never/Rarely: 60.1% vs. 23.4%; Sometimes: 21.8% vs. 12.8%; Often/Always: 18.1% vs. 63.8%, respectively; *p* < 0.001). A higher proportion of participants engaging in six or more family protection actions were recruited in Nickerie relative to Paramaribo (89.4% vs. 36.7%, respectively; *p* < 0.001). Mothers recruited in Paramaribo were on average older than those recruited in Nickerie (mean 34.4 years, [SD 6.4] vs. mean 31.2 years [SD 5.8], respectively; *p* = 0.004). More unvaccinated mothers were recruited in Paramaribo than in Nickerie (47.3% vs. 25.5%, respectively; *p* = 0.011). The estimated SARS-CoV-2 seroprevalence in C2 was higher than in C1 (93.3% [95% CI: 89.9–95.6%] vs. 33.8% [95% CI: 28.5–39.6%], respectively; Table 2). No differences were found in the estimated seroprevalence from collection time points by age group, gender, ethnicity, or recruitment region.

### 3.2. SARS-CoV-2 Seroprevalence

**Figure 3:**
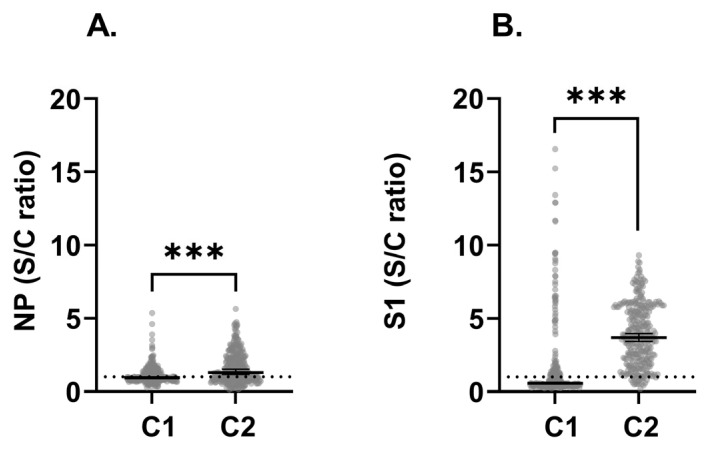
Children’s IgG antibody response to SARS-CoV-2 NP and S1 cross-sectionally and longitudinally. Total IgG anti-NP (**A**) or IgG anti–S1 (**B**) OD450 nm were measured by in-house ELISA assay from DBS eluted samples collected at the first time point C1 (N = 275) and the second time point C2 (N = 298). Samples with a ratio >1 were identified as positive and ≤1 as negative. The dotted lines represent the cut-off of 1. The blank background was subtracted from controls and samples. The cut-off for each plate was calculated as the adjusted average OD450 nm of the negative control + 3x(SD negative control). Data analysis is shown as scatter plots with symbols representing individual participants and bars representing the median ±95% CI. Statistical significance was calculated using the two-sided Mann–Whitney U test or the Wilcoxon matched pairs test. Significant *p*-values (*p* *** < 0.001) were obtained. C1, collection time point 1 from 3 January 2021 to 19 September 2022; C2, collection time point 2 from 21 February 2023 to 22 April 2023; S1, spike domain 1; NP, nucleoprotein; S/C, sample to cut-off ratio.

**Figure 4:**
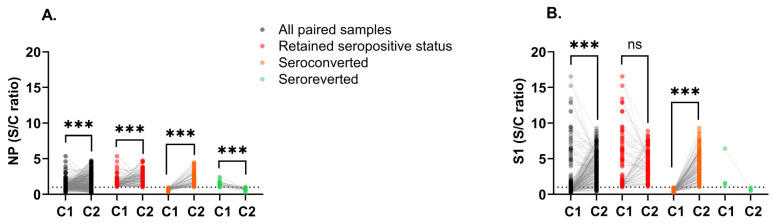
Longitudinal antibody response to SARS-CoV-2 NP and S1. (**A**,**B**) Scatter plot of matched pairs by collection time. The S/C ratio was calculated as the sample adjusted OD450 nm/cut-off for the plate. Samples with a ratio >1 were identified as positive and ≤1 as negative. The dotted lines represent the cut-off of 1. The blank background was subtracted from controls and samples. The cut-off for each plate was calculated as the adjusted average OD450 nm of the negative control + 3x(SD negative control). Data analysis is shown as scatter plots with symbols representing individual participants and bars representing the median ± 95% CI. Statistical significance was calculated using the two-sided Mann–Whitney U test or the Wilcoxon matched pairs test. Significant *p*-values (*p* *** < 0.001) were obtained. C1, collection time point 1 from 3 January 2021 to 19 September 2022; C2, collection time point 2 from 21 February 2023 to 22 April 2023; S1, spike domain 1; NP, nucleoprotein; ns, not significant; S/C, sample to cut-off ratio.

**Figure A2:**
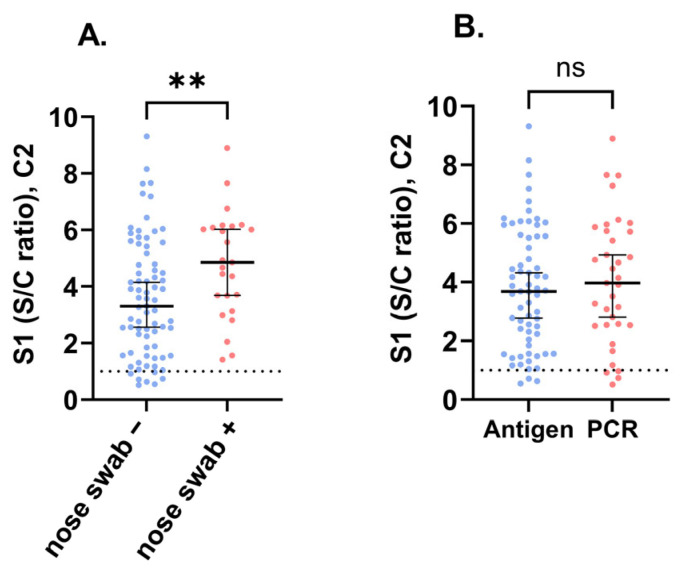
Total IgG SARS-CoV-2 S1 S/C ratios of children who were tested for COVID-19 at any point in time. Children nose swab testing at any point in time was reported by their mothers via questionnaire at C2. (**A**) Nose swab negative and positive results (blue N = 75 and red N = 25, respectively). (**B**) Type of nose swab test: antigen and PCR tests (blue N = 65 and red N = 35, respectively). Data analysis is shown as scatter plots with symbols representing individual participants and bars representing the median ±95% CI. Statistical significance was calculated using the two-sided, Mann–Whitney U test. A significant *p* value (*p* ** < 0.005) was obtained. C2, collection time point 2 from 21 February 2023 to 22 April 2023; S1, spike domain 1; ns, not significant; S/C, sample to cut-off ratio; PCR, Polymerase chain reaction test; CI, confidence intervals.

**Figure A3:**
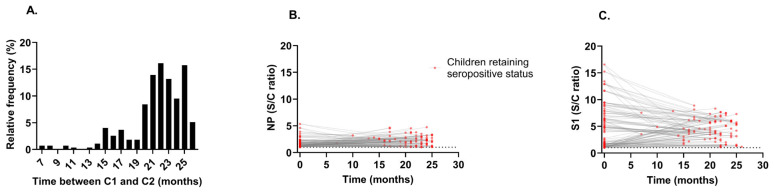
Time (months) between DBS collection C1 and C2. (**A**) Histogram showing the time in months of DBS collection time points. The mean time between collections of all paired samples was 21.5 months (standard deviation (SD) 3.6, 95% CI 21.1–21.9). IgG anti-NP (**B**) or anti-S1 (**C**) S/C ratios of seropositive children in C1 (month 0) that retained seropositive status in C2. Statistical analysis was performed in GraphPad Prism 10.0.3. C1, collection time point 1 from 3 January 2021 to 19 September 2022; C2, collection time point 2 from 21 February 2023 to 22 April 2023; NP, nucleoprotein; S1, spike domain 1; n, sample size; S/C, sample to cut-off ratio; IgG, immunoglobulin G; CI, confidence intervals.

**Figure A4:**
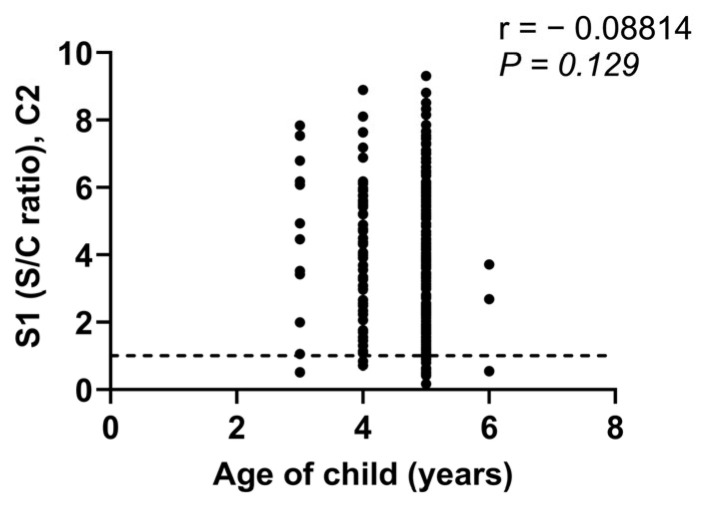
Levels of total IgG anti-S1 by age of children in C2. The S/C ratio was calculated as the sample adjusted OD450 nm/cut-off for the plate. Samples with a ratio >1 were identified as positive and ≤1 as negative. The dashed lines represent the cut-off of 1. A Spearman correlation was performed in GraphPad Prism 10.0.3. r, rho; *p*, *p*-value; C2, collection time point 2 from 21 February 2023 to 22 April 2023; S1, spike domain 1; S/C, sample to cut-off ratio.

**Figure A5:**
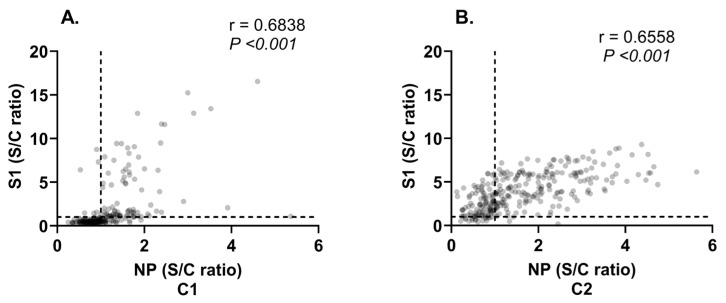
S/C ratios of total IgG anti-NP and S1 from eluted DBS samples of children from two collection time points. Spearman correlation analysis of total IgG anti-SARS-CoV-2 NP and/or S1 [C1: N = 275; C2: N = 298] (**A**,**B**). The S/C ratio was calculated as the sample adjusted OD450 nm/cut-off for the plate. Samples with a ratio >1 were identified as positive and ≤1 as negative. The dashed lines represent the cut-off of 1. Statistical analysis performed in GraphPad Prism 10.0.3. r, rho; *p*, *p*-value; C1, collection time point 1 from 3 January 2021 to 19 September 2022; C2, collection time point 2 from 21 February 2023 to 22 April 2023; NP, nucleoprotein; S1, spike domain 1; S/C, sample to cut-off ratio.

Of a total of 100 children, 25 tested positive for COVID-19 by diagnostic test (PCR or antigen), of which 18 were tested after C1, and 24 were symptomatic as reported by their mothers (Table A3, Figure A2). The mean time between collection time points C1 and C2 was 21.5 months (Figure A3A). Children had higher levels of antibodies in C2, suggesting an increased response to the virus over collection times. We observed significantly higher IgG anti-NP (*p* < 0.001) and S1 (*p* < 0.001) S/C ratios in C2 compared to C1 (Figure 3A and Figure 3B, respectively). Analysis of paired samples from 273 children showed a significant increase in antibody levels against SARS-CoV-2 NP among children who retained seropositive status (*p* < 0.001) and seroconverted (*p* < 0.001) by C2 (Figure 4A). Children with seroreverted anti-NP status had significantly higher antibody levels in C1. A significant increase in antibody levels against SARS-CoV-2 S1 was observed among children who seroconverted (*p* < 0.001) by C2 (Figure 4B). About 34.4% of children seroconverted for anti-NP between C1 and C2, 29.3% retained seropositive status, 23.8% retained seronegative status, and 12.5% seroreverted (Table 3). In contrast, 60.1% of children seroconverted for anti-S1, 32.6% retained seropositive status, 5.9% retained seronegative status, and 1.5% seroreverted. No correlation was found between anti-S1 S/C ratios in C2 by the age of children (Figure A4, *p* = 0.129). However, there were significant positive correlations between IgG anti-NP and S1 S/C ratios cross-sectionally (Figure A5A and Figure A5B: r = 0.6838, *p* < 0.001 and r = 0.6558, *p* < 0.001, respectively). Although antibody S/C ratios for NP and S1 increased in C2, anti-S1 was higher than NP (Figure A5B). Cross-tables, Cohen’s kappa test, sensitivity, and specificity of serology results are shown in Table A4, Equations (A1) and (A2), Table A5, and Equations (A3) and (A4).

### 3.3. Determinants of SARS-CoV-2 Seropositivity in Children

**Figure A6:**
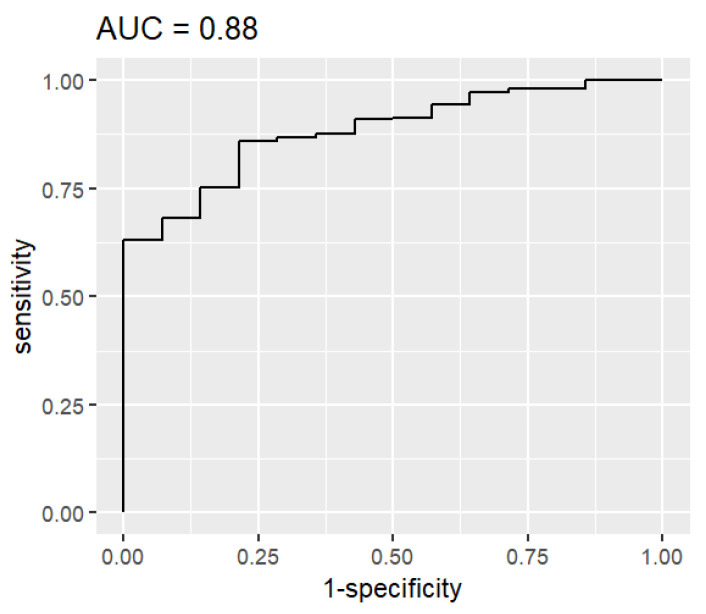
AUC and ROC analysis for the final model. AUC, Area Under the Curve; ROC, Receiver Operating Characteristic curve.

Only the age of the mother and household size were univariately associated with SARS-CoV-2 seroprevalence (Table A6). The variables included in the final model were the age of the mother, household size, COVID-19 diagnosis by a healthcare provider, social activities of children during the COVID-19 pandemic, use of masks by children, distancing practices by children, and the mother’s COVID-19 vaccination (Table 4). The age of the mother, household size, and mother’s COVID-19 vaccination were significant predictors of children’s SARS-CoV-2 seropositivity in the final model after adjustment for covariates. A lower seropositivity likelihood was observed among children with older mothers above 33 years (adjusted odds ratio (aOR) 0.88 [95% CI: 0.78–0.97, *p* = 0.017]). A lower seropositivity likelihood was observed among children living in households with 5 or more members (5 or more members: aOR 0.11 [95% CI: 0.01–0.54, *p* = 0.017]). Children from unvaccinated mothers were 6.11 times more likely to be seropositive (not vaccinated: aOR 6.11 [95% CI: 1.47–34.46, *p* = 0.022]). The results from the final model were validated by assessing its accuracy (Table A7 and Equation (A5)), sensitivity and specificity (Equations (A6) and (A7)), AUC and ROC curve (Figure A6), VIF (Table A8), and goodness of fit (Appendix A.1.5).

### 3.4. Correlations Between Variables in the Final Model

We tested correlations between the predictors of the final model from Table 4. Maternal COVID-19 vaccination was significantly correlated to COVID-19-positive household contacts (χ^2^: *p* = 0.003). Distancing practiced by the child in social activities during the COVID-19 pandemic was significantly correlated to maternal COVID-19 vaccination (χ^2^: *p* = 0.029). Other correlations were not statistically significant.

### 3.5. Interaction Effects

The LRT comparing binary logistic regression models with and without the interaction between the mother’s age and the child’s distancing practice in social activities was statistically significant (*p* = 0.047). Children of mothers aged 33 or older practiced social distancing more frequently during social activities and were less likely to be infected. However, this interaction term was not included in the final model, as it influenced model stability (wide 95% CI) and introduced multicollinearity (VIFs > 10).

## 4. Discussion

### 4.1. SARS-CoV-2 Seroprevalence of Children Within the CCREOH Cohort

This is one of the first studies to assess SARS-CoV-2 seroprevalence among young children in Suriname and COVID-19 behaviors. The seroprevalence increased from 33.8% (95% CI: 28.5–39.6%) in C1 to 93.3% (95% CI: 89.9–95.6%) in C2, supporting our hypothesis. Consistent with our results, in Latin America and the Caribbean, the SARS-CoV-2 seroprevalence of children was 3.56% (95% CI: 2.09–5.07%) and 21.87% (95% CI: 3.90–48.67%) in the first and fourth pandemic waves, respectively [3]. A study in Nicaragua found high SARS-CoV-2 infection rates in 51.6% of children between the ages of 0 and 14 years [8]. Meanwhile, in the Brazilian Amazon, the seroprevalence of 297 children was 45.0% (95% CI: 41.2–48.9%) at the age of 5 years [2]. Only 15 of these children were previously diagnosed with COVID-19, as reported by their mothers or caregivers. In our study, 25 children were diagnosed with COVID-19 in a cohort of 300, highlighting the underdiagnosis of most pediatric infections. Of 273 children, most seroconverted to anti-S1 antibodies, suggesting that these last longer and better reflect exposure levels than anti-NP antibodies, consistent with another study [22].

### 4.2. Behavioral and Social Determinants of SARS-CoV-2 Seropositivity

Age of mother, household size, and maternal COVID-19 vaccination were significant predictors of pediatric SARS-CoV-2 seroprevalence. Children of older mothers and vaccinated mothers, as well as those living in households of five or more members were less likely to get infected and thus were better protected. In contrast, children with unvaccinated mothers were more likely to be infected with SARS-CoV-2. Consistent with our results, the study by Hayek et al. [23] found that SARS-CoV-2 vaccination of parents reduces the risk of infection in their unvaccinated children within the household. Having one vaccinated parent reduced the risk of child infection by 26% and 20.8% during the Alpha and Delta waves, respectively; the risk was further reduced by 71.7% and 58.1% when both parents were vaccinated. Other studies show that vaccination of parents reduced the risk of hospitalization in children under five during the Delta and Omicron waves [24]. A study of children under age six years old in a Swiss canton found that those living in households of five or more members were less likely to be seropositive [25]. For these children, the likelihood of seropositivity increased with the number of household members aged 12 or older who had confirmed COVID-19 PCR tests. Children living in large households may be protected if they are less frequently in contact with COVID-19-infected parents or household members.

### 4.3. Correlations Among the Predictors in the Final Model

Maternal COVID-19 vaccination and COVID-19-positive household contacts were significantly correlated, suggesting that mothers who feel at risk of infection from household transmission may decide to vaccinate themselves and protect their children from exposure. Child distancing practices and maternal vaccination were correlated, suggesting that vaccinated mothers were more protective of their children by facilitating more frequent distancing practices.

### 4.4. COVID-19 Prevention Practices

In Suriname, there was no policy for a minimum age for mask-wearing. However, parents would mask their children, particularly if the child presented COVID-like symptoms. We identified a caveat from our findings. There were no differences in mask use between the Paramaribo and Nickerie recruitment regions after 21.5 months on average, but mask use did not necessarily slow the infection rate. Distancing practices among children during social activities were significantly correlated with recruitment region: children from Paramaribo complied less frequently with distancing than those recruited in Nickerie. Young children are more physically active and play with other children, which is why wearing a mask and practicing distancing might be challenging for them. In Suriname, children under 4 years are usually under the care of family members, neighbors, or friends, and prevention might be encouraged when the child or the caregiver is infected, in line with other studies [3,25]. Across districts, the most frequent protection actions practiced by families were those recommended by authorities [26] and the country’s Mother Health in Action, or MoHanA, program [27], which included wearing masks, washing hands with soap, distancing 1.5 m (6 ft) apart from people, and using hand sanitizer. Reports from Suriname emphasized the lack of compliance with social prevention measures during or late in the pandemic, suggesting possible recall or social desirability bias in our study population [28,29]. In addition, after random sampling, we obtained relatively few participants from Coronie, Para, Commewijne, and Saramacca, hindering the evaluation of the true extent of compliance or non-compliance with social prevention measures.

### 4.5. Limitations of the Study

This study has some limitations. Self-reported responses in the questionnaire were conditioned to social desirability and recall bias, especially with retrospective questions. This leads to an underestimation of COVID-19 exposure among children and family members and an overestimation of compliance with prevention practices. Our sample population included mother and child dyads from the prospective CCREOH cohort, and questionnaire responses do not account for both parents or all direct caregivers. The date of diagnostic testing for children was approximate due to the time elapsed between the test and data collection. Our study was designed for questionnaire data collection in C2, which explains the absence of C1 questionnaires. Thus, our ability to accurately determine the change in COVID-19-related behaviors and their association with seroprevalence was hindered. The questionnaire was not piloted to minimize recall bias. The random sampling within the CCREOH cohort is a strength. However, low participation in Coronie, Para, and Saramacca limits the generalizability of the findings to the Suriname population. The specificity of the final model was low, a common feature of population-level diagnostics. In contrast, the sensitivity was high. For our model, we accounted for a sample population of 219 seropositive and 14 seronegative children. Consequently, model stability was compromised by bias arising from low counts of seronegative children in variable categories. This issue is most notable with one seronegative child recruited in Nickerie and three seronegative children from unvaccinated mothers (Table A6). As a result, we obtained wide confidence intervals for the adjusted odds ratios of the final model. Removing the interaction term reduced multicollinearity, thereby stabilizing the estimated regression coefficients. In turn, the confidence intervals for the adjusted odds ratios are narrower. Another limitation is the reduced sample size after data cleaning for regression analysis.

Our study is limited to maternal vaccination status and does not assess COVID-19 vaccination status for each family member or external caregiver. The age and prior COVID-19 infection status of each family member were not evaluated. Our findings are limited to young children aged 6 years or less and cannot be generalized to older children in the Suriname population. Family protection actions during the COVID-19 pandemic were not included in the regression analysis due to mixed responses.

## 5. Conclusions

This study found a striking increase in SARS-CoV-2 seroprevalence from 33.8% to 93.3% over a 21.5 month follow-up, reflecting multiple exposures. Most children were never tested for COVID-19, highlighting that many potential cases of infection were undiagnosed and unreported. Children of non-immunized mothers were more likely to be seropositive by 2023, highlighting that maternal COVID-19 vaccination confers protection against COVID-19 exposure in children. Regional interventions should be tailored for children of unvaccinated mothers. We recommend future studies evaluating the cultural and behavioral context of living arrangements in Suriname and their association with COVID-19 household exposure and transmission. We also recommend assessing additional risk factors found to be significantly associated with COVID-19 prevalence in Latin America and the Caribbean, and LMICs by other studies, including household income [3], poverty [2,3,12], food insecurity [2], a detailed history of infection and vaccination of children and adults within the household [2], disease and vaccine risk awareness and perceptions [12], trust in public health communications [29], and seasonality [30].

## Figures and Tables

**Table 1:** Comparison of demographic characteristics of the study population by recruitment region at C2.

Variables	Overall (N = 235)	Paramaribo Region (N = 188)	Nickerie Region (N = 47)	*p*-Value
Age of mother in years, mean	33.7	34.4	31.2	0.004
Age of child in years, mean	4.8	4.8	4.5	<0.001
Gender				1.000
Female	132	106 (56.4%)	26 (55.3%)	
Male	103	82 (43.6%)	21 (44.7%)	
Ethnicity				<0.001
Asian descent	67	43 (22.9%)	24 (51.1%)	
African descent	87	84 (44.7%)	3 (6.4%)	
Other (Indigenous/Amerindian, Mixed, Caucasian, Other)	81	61 (32.4%)	20 (42.6%)	
Household size				0.412
4 or fewer	105	87 (46.3%)	18 (38.3%)	
5 or more	130	101 (53.7%)	29 (61.7%)	
Level of education (mother)				1.000
None/primary/lower secondary	124	99 (52.7%)	25 (53.2%)	
Technical vocational/secondary/higher education (masters/bachelor)/other	111	89 (47.3%)	22 (46.8%)	
Employment				0.065
Employed	168	140 (74.5%)	28 (59.6%)	
Unemployed	67	48 (25.5%)	19 (40.4%)	
Family protection actions during the COVID-19 pandemic				0.001
5 or fewer	74	69 (36.7%)	5 (10.6%)	
6 or more	161	119 (63.3%)	42 (89.4%)	
Social activities of children during the COVID-19 pandemic				<0.001
3 or fewer	118	82 (43.6%)	36 (76.6%)	
4 or more	117	106 (56.4%)	11 (23.4%)	
Use of mask by child in social activities				0.277
Never/rarely	35	27 (14.4%)	8 (17.0%)	
Sometimes	38	34 (18.1%)	4 (8.5%)	
Often/always	162	127 (67.6%)	35 (74.5%)	
Distancing practice by child in social activities				<0.001
Never/rarely	124	113 (60.1%)	11 (23.4%)	
Sometimes	47	41 (21.8%)	6 (12.8%)	
Often/always	64	34 (18.1%)	30 (63.8%)	
COVID-19 diagnosis of the child by a healthcare provider				1.000
No	166	133 (70.7%)	33 (70.2%)	
Yes	69	55 (29.3)	14 (29.8)	
COVID-19-positive household contacts				0.181
No positives	117	89 (47.3%)	28 (59.6%)	
Positives	118	99 (52.7%)	19 (40.4%)	
Mother COVID-19 vaccination				0.011
Vaccinated	134	99 (52.7%)	35 (74.5%)	
Not vaccinated	101	89 (47.3%)	12 (25.5%)	

C2, collection time point 2 from 21 February 2023 to 22 April 2023; N, sample size. Continuous variables are represented as a mean. Categorical variables are represented with counts and/or percentages.

**Table 2:** SARS-CoV-2 S1 antibody seroprevalence estimates and demographics of children at C1 and C2.

Collection Time	Variables	Overall, N (%)	Seropositive, N	Seroprevalence Estimates % (95% CI) ^a^	OR (95% CI)	*p*-Value
C1	Total	275 (100.0)	93	33.8 (28.5–39.6)	—	—
	Age of child in years ^b^					
	1–2	53 (19.3)	17	32.1 (21.1–45.5)	Ref.	
	3–4	222 (80.7)	76	34.2 (28.3–40.7)	1.10 (0.59–2.13)	0.77
	Gender					
	Female	148 (53.8)	51	34.5 (27.3–42.4)	Ref.	
	Male	127 (46.2)	42	33.1 (25.5–41.6)	0.94 (0.57–1.55)	0.81
	Ethnicity					
	Asian descent	96 (34.9)	33	34.4 (25.6–44.3)	Ref.	
	African descent	92 (33.5)	35	38.0 (28.8–48.3)	1.17 (0.65–2.13)	0.60
	Other (Indigenous/Amerindian, Mixed, Caucasian, Other)	87 (31.6)	25	28.7 (20.3–39.0)	0.77 (0.41–1.44)	0.41
C2	Total	298 (100)	278	93.3 (89.9–95.6)	—	—
	Age of child in years ^b^					
	3–4	78 (26.2)	74	94.9 (87.5–98.0)	Ref.	
	5–6	220 (73.8)	204	92.7 (88.5–95.5)	0.69 (0.19–1.95)	0.52
	Gender					
	Female	161 (54.0)	152	94.4 (89.7–97.0)	Ref.	
	Male	137 (46.0)	126	92.0 (86.2–95.5)	0.68 (0.27–1.69)	0.40
	Ethnicity					
	Asian descent	107 (35.9)	101	94.4 (88.3–97.4)	Ref.	
	African descent	93 (31.2)	86	92.5 (85.3–96.3)	0.73 (0.23–2.28)	0.58
	Other (Indigenous/Amerindian, Mixed, Caucasian, Other)	98 (32.9)	91	92.9 (86.0–96.5)	0.77 (0.24–2.41)	0.65
	Recruitment region					
	Paramaribo region	208 (69.8)	194	93.3 (89.0–96.0)	Ref.	
	Nickerie region	90 (30.2)	84	93.3 (86.2–97.0)	1.01 (0.39–2.93)	0.98

C1, collection time point 1 from 3 January 2021 to 19 September 2022; C2, collection time point 2 from 21 February 2023 to 22 April 2023; N, sample size; Ref, reference category; OR, unadjusted odds ratios; CI, confidence intervals. ^a^ Wilson’s scores; ^b^ Age of children as a categorical variable.

**Table 3:** Longitudinal SARS-CoV-2 seroprevalence of children with paired samples.

Seroprevalence Change	NP (N = 273)	S1 (N = 273)
Retained seropositive status	80 (29.3%)	89 (32.6%)
Seroconverted	94 (34.4%)	164 (60.1%)
Seroreverted	34 (12.5%)	4 (1.5%)
Retained seronegative status	65 (23.8%)	16 (5.9%)

**Table 4:** Adjusted odds ratios and confidence intervals for predictors of SARS-CoV-2 seroprevalence of children at C2.

Variables	aOR	95% CI	*p*-Value
Age of mother in years	0.88	0.78–0.97	0.017
Recruitment region			
Paramaribo region	Ref.	—	—
Nickerie region	6.34	0.84–134.78	0.119
Household size			
4 or fewer	Ref.	—	—
5 or more	0.11	0.01–0.54	0.017
Social activities of children during the COVID-19 pandemic			
3 or fewer	Ref.	—	—
4 or more	0.42	0.10–1.52	0.198
Use of masks by children in social activities			
Never/Rarely	Ref.	—	—
Sometimes	1.41	0.12–15.70	0.774
Often/Always	2.33	0.26–14.95	0.392
Distancing practice by child in social activities			
Never/Rarely	Ref.	—	—
Sometimes	0.52	0.09–3.52	0.472
Often/Always	0.28	0.05–1.47	0.131
COVID-19 diagnosis by a healthcare provider			
No	Ref.	—	—
Yes	1.79	0.42–10.95	0.473
COVID-19-positive household contacts			
No positives	Ref.	—	—
Positives	3.34	0.80–17.12	0.115
Mother’s COVID-19 vaccination			
Vaccinated	Ref.	—	—
Not vaccinated	6.11	1.47–34.46	0.022

Ref, reference category; aOR, adjusted odds ratios; CI, confidence intervals. aORs estimated from the final binary logistic regression model are shown.

## Data Availability

The data presented in this study are available on request from the corresponding author with prior consultation with the Office of Sponsored Programs of the University of Pittsburgh. The R code used for data analyses is provided at https://github.com/dbarreto967/SARS-CoV-2-seroprevalence-CCREOH/tree/main (accessed on 19 March 2026).

## Supplementary Materials

The following supporting information can be downloaded at: https://www.mdpi.com/article/10.3390/children13040493/s1, Questionnaire provided in File S1: Impacts of COVID-19 on Surinamese children.

## Abbreviations

The following abbreviations are used in this manuscript:

CCREOH	Caribbean Consortium for Research in Environmental and Occupational Health
SARS-CoV-2	Severe Acute Respiratory Syndrome Coronavirus 2
COVID-19	coronavirus disease 2019
IgG	immunoglobulin G
S1	spike domain 1
NP	nucleoprotein
C1	collection time point 1
C2	collection time point 2
DBS	dried blood spots
S/C	sample to cut-off ratio
ELISAs	Enzyme-linked immunosorbent assays
PCR	Polymerase chain reaction
IRB	Institutional Review Board
REDCap	Research Electronic Data Capture
CTSI	Clinical and Translational Science Institute
χ^2^	Pearson’s Chi-squared test
CI	Confidence Intervals
LRT	Likelihood Ratio Test
SD	Standard Deviation
OR	odds ratio
aOR	adjusted odds ratio
OD	Optical Density
AUC	Area Under the Curve
ROC	Receiver Operating Characteristic
VIF	Variance Inflation Factor

## Appendix A

**Table A1:** Aggregated variables.

Variables	Categories (Description)	Reason for Aggregating/Recoding
Ethnicity	Asian descent (Chinese, Hindustani, and Javanese) African descent (Creole and Maroon) Other (Indigenous/Amerindian, Mixed, Caucasian, Other)	The variable from a multiple-choice question. Aggregated for use in statistical analyses.
Household size	Four or fewer (ranging from 2 to 4 members) Five or more (ranging from 5 to 12 members)	Recoded as a categorical variable for use in statistical analyses. Household size, as a continuous variable, lacks a unit of measurement.
Level of education (mother)	None/primary/lower secondary Technical vocational/secondary/Higher education (Masters/Bachelor)/other	Quasi-complete separation, i.e., issues with estimability. The “Higher education (Masters/Bachelor)/other” level had 0 counts in the seronegative level of the outcome “Total IgG anti-S1 Seroprevalence of 2023 (C2)”. The regression model estimates were inflated. The solution was to combine the levels “Technical vocational/secondary” and “Higher education (Masters/Bachelor)/other “.
Employment	Employed (employed for wages and self-employed) Unemployed (unemployed—looking for work, unemployed—not looking for work, homemaker, student, retired, unable to work due to disability, other, and mixed)	The variable from a multiple-choice question. Aggregated for use in statistical analyses.
Family protection actions during the COVID-19 pandemic	Five or fewer (ranging from 3 to 5 actions) Six or more (ranging from 6 to 8 actions)	The variable originated from a multiple-choice question. Aggregated for use in statistical analyses.
Social activities of children during the COVID-19 pandemic	None Three or fewer (ranging from 1 to 3 activities) Four or more (ranging from 1 to 10 activities)	The variable originated from a multiple-choice question. Aggregated for use in statistical analyses.

**Table A2:** Comparison between models with main effects (no interaction) and models with interactions before binary logistic regression modeling.

Models with Main Effects	Models with Interaction	*p*-Value ^a^
age_motherc + region_residence	age_motherc * region_residence	0.677
age_motherc + distancing3	age_motherc * distancing3	0.034
age_motherc + mask4	age_motherc * mask4	0.063
age_motherc + pos_household	age_motherc * pos_household	0.058
mother_vaccine + age_motherc	mother_vaccine * age_motherc	0.438
mother_vaccine + age_childc	mother_vaccine * age_childc	0.208
mother_vaccine + gender_child	mother_vaccine * gender_child	0.720
mother_vaccine + ethnicity3	mother_vaccine * ethnicity3	0.257
mother_vaccine + region_residence	mother_vaccine * region_residence	0.664
mother_vaccine + num_house	mother_vaccine * num_house	0.372
mother_vaccine + education3	mother_vaccine * education3	0.770
mother_vaccine + employment2	mother_vaccine * employment2	0.194
mother_vaccine + childactivities3	mother_vaccine * childactivities3	0.084
mother_vaccine + mask4	mother_vaccine * mask4	0.096
mother_vaccine + distancing3	mother_vaccine * distancing3	0.715
mother_vaccine + COVID_diagnosis	mother_vaccine * COVID_diagnosis	0.285
mother_vaccine + pos_household	mother_vaccine * pos_household	0.282
age_motherc + num_house	age_motherc * num_house	0.054
num_house + pos_household	num_house * pos_household	0.180

The age of the mother and the age of the child are continuous variables and are centered. ^a^ *p*-value of the likelihood ratio test with a chosen significance of 0.05. Symbols “+” and “*” indicate the main effects and the interaction, respectively.

**Table A3:** Types of nose swab from children tested for COVID-19 at any point in time.

Variables	Overall, N (%)	Nose Swab Negative, N (%)	Nose Swab Positive, N (%)
Total	100 (100.0)	75 (75.0)	25 (25.0)
Type of nose swab test			
PCR	35 (35.0)	27 (36.0)	8 (32.0)
Antigen	65 (65.0)	48 (64.0)	17 (68.0)

N, sample size.

**Table A4:** Cross table for Cohen’s kappa inter-reliability test of SARS-CoV-2 antibody seroprevalence in 2021–2022 (C1).

		Anti-S1 Seroprevalence	
Anti-NP Seroprevalence	Overall, N (%)	Seronegative, N (%)	Seropositive, N (%)
**Total**	275 (100.0)	182 (66.2)	93 (33.8)
Seronegative	161 (58.6)	155 (96.3)	6 (3.7)
Seropositive	114 (41.5)	27 (23.7)	87 (76.3)

C1, collection time point 1 from 3 January 2021 to 19 September 2022; N, sample size; NP, nucleoprotein; S1, spike domain 1.

Sensitivity = True positives/(true positives + false negatives) × 100 = 87/(87 + 6) × 100 = 93.5% (A1)

Sensitivity refers to the percentage of seropositive children who are correctly identified as seropositive. Where true positives are children correctly diagnosed as seropositive, and false negatives are children incorrectly diagnosed as seronegative.

Specificity = True negatives/(true negatives + false positives) × 100 = 155/(155 + 27) × 100 = 85.2% (A2)

Specificity refers to the percentage of seronegative children who are correctly identified as seronegative. Where true negatives are children correctly diagnosed as seronegative and false positives are children incorrectly diagnosed as seropositive.

The Cohen’s kappa test is statistically significant (*p* < 0.001). The kappa value of 0.746 indicates a moderate inter-rater reliability between IgG anti-NP and S1 results of C1. The test was performed in R v4.4.2.

**Table A5:** Crosstable for Cohen’s kappa inter-reliability test of SARS-CoV-2 antibody seroprevalence in 2023 (C2).

		Anti-S1 Seroprevalence	
Anti-NP Seroprevalence	Overall, N (%)	Seronegative, N (%)	Seropositive, N (%)
**Total**	298 (100.0)	20 (6.7)	278 (93.3)
Seronegative	109 (36.6)	17 (15.6)	92 (84.4)
Seropositive	189 (63.4)	3 (1.6)	186 (98.4)

C2, collection time point 2 from 21 February 2023 to 22 April 2023; N, sample size; NP, nucleoprotein; S1, spike domain 1.

Sensitivity = True positives/(true positives + false negatives) × 100 = 186/(186 + 92) × 100 = 66.9% (A3)

Sensitivity refers to the percentage of seropositive children who are correctly identified as seropositive. Where true positives are children correctly diagnosed as seropositive, and false negatives are children incorrectly diagnosed as seronegative.

Specificity = True negatives/(true negatives + false positives) × 100 = 17/(17 + 3) × 100 = 85.0% (A4)

Specificity refers to the percentage of seronegative children who are correctly identified as seronegative. True negatives are children correctly diagnosed as seronegative and false positives are children incorrectly diagnosed as seropositive.

The Cohen’s kappa test is statistically significant (*p* < 0.001). The kappa value of 0.169 indicates none inter-rater reliability between IgG anti-NP and anti-S1 results of C2. The test was performed in R 4.4.2.

**Table A6:** Summary of the data and univariate regression analysis.

Variables	Overall (N = 233)	Seronegative (N = 14)	Seropositive (N = 219)	Univariate Analysis, OR (95% CI)	*p*-Value ^a^
Age of mother in years, mean (SD) ^b^	33.8 (6.4) ^b^	39.4 (6.8) ^b^	33.4 (6.2) ^b^	0.86 (0.78–0.94)	0.001
Age of child in years, mean (SD) ^b^	4.8 (0.5) ^b^	4.8 (0.7) ^b^	4.8 (0.5) ^b^	0.93 (0.27–2.48)	0.894
Gender					
Female	132 (56.7%)	6 (42.9%)	126 (57.5%)	Ref.	
Male	101 (43.4%)	8 (57.1%)	93 (42.5%)	0.55 (0.18–1.64)	0.288
Ethnicity					
Asian descent	67 (28.8%)	3 (21.4%)	64 (29.2%)	Ref.	
African descent	86 (36.9%)	6 (42.9%)	80 (36.5%)	0.63 (0.13–2.47)	0.518
Other (Indigenous/Amerindian, Mixed, Caucasian, Other)	80 (34.3%)	5 (35.7%)	75 (34.2%)	0.70 (0.14–2.98)	0.639
Recruitment region					
Paramaribo region	187 (80.3%)	13 (92.9%)	174 (79.5%)	Ref.	
Nickerie region	46 (19.7%)	1 (7.1%)	45 (20.5%)	3.36 (0.64–61.88)	0.249
Household size					
4 or fewer	103 (44.2%)	2 (14.3%)	101 (46.1%)	Ref.	
5 or more	130 (55.8%)	12 (85.7%)	118 (53.9%)	0.19 (0.03–0.74)	0.035
Level of education (mother)					
None/primary/lower secondary	124 (53.2%)	8 (57.1%)	130 (59.4%)	Ref.	
Technical vocational/secondary/higher education (masters/bachelor)/other	109 (46.8%)	6 (42.9%)	103 (47.0%)	1.18 (0.40–3.70)	0.762
Employment					
Employed	167 (71.7%)	10 (71.4%)	157 (71.7%)	Ref.	
Unemployed	66 (28.3%)	4 (28.6%)	62 (28.3%)	0.99 (0.32–3.71)	0.983
Social activities of children during the COVID-19 pandemic					
3 or fewer	117 (50.2%)	5 (35.7%)	112 (51.1%)	Ref.	
4 or more	116 (49.8%)	9 (64.3%)	107 (48.9%)	0.53 (0.16–1.59)	0.270
Use of mask by child in social activities					
Never/rarely	35 (15.0%)	2 (14.3%)	33 (15.1%)	Ref.	
Sometimes	38 (16.3%)	3 (21.4%)	35 (16.0%)	0.71 (0.09–4.52)	0.714
Often/always	160 (68.7%)	9 (64.3%)	151 (68.9%)	1.02 (0.15–4.18)	0.983
Distancing practice by child in social activities					
Never/rarely	123 (52.8%)	6 (42.9%)	117 (53.4%)	Ref.	
Sometimes	47 (20.2%)	3 (21.4%)	44 (20.1%)	0.75 (0.19–3.68)	0.696
Often/always	63 (27.0%)	5 (35.7%)	58 (26.5%)	0.59 (0.17–2.14)	0.407
COVID-19 diagnosis by healthcare provider					
No	164 (70.4%)	11 (78.6%)	153 (69.9%)	Ref.	
Yes	69 (29.6%)	3 (21.4%)	66 (30.1%)	1.58 (0.48–7.16)	0.492
COVID-19-positive household contacts					
No positives	116 (49.8%)	10 (71.4%)	106 (48.4%)	Ref.	
Positives	117 (50.2%)	4 (28.6%)	113 (51.6%)	2.67 (0.86–9.95)	0.106
Mother COVID-19 vaccination					
Vaccinated	134 (57.5%)	11 (78.6%)	123 (56.2%)	Ref.	
Not vaccinated	99 (42.5%)	3 (21.4%)	96 (43.8%)	2.86 (0.87–12.91)	0.114

N, sample size; SD, standard deviation; Ref, reference category; OR, unadjusted odds ratios; CI, confidence intervals. ^a^ *p*-values of univariate analysis; ^b^ continuous variables are represented as mean (SD) and centered for regression analyses. Categorical variables are represented as N (%).

### Appendix A.1. Validation of the Final Model

#### Appendix A.1.1. Final Model Training and Accuracy

Steps:

1. Train the model on the initial dataset.
2. Test the model on the same dataset.

- Create a vector of predicted outcomes.
- Establish a threshold for the probability of a child being seropositive. Here, we propose a default threshold of 50%, i.e., if the probability of a child being seropositive is below 50%, then the child is seronegative.

3. Compare the predicted outcomes made by the model to the observed outcomes.

**Table A7:** Observed vs. predicted values from C2.

	Predicted	
Observed	Seropositive, N	Seronegative, N
Seropositive	217	2
Seronegative	12	2

C2, Collection time point 2 from 21 February 2023 to 22 April 2023; N, sample size.

Accuracy = Sum of the correct predictions/total number of predictions = 94.0% (A5)

Accuracy refers to the percentage of correct predictions (or accuracy). Accuracy was calculated in R 4.4.2.

#### Appendix A.1.2. Sensitivity and Specificity of the Final Model

Sensitivity = True positives/(true positives + false negatives) × 100 = 217/(217 + 2) × 100 = 99.1% (A6)

Specificity = True negatives/(true negatives + false positives) × 100 = 2/(2 + 12) × 100 = 14.3% (A7)

Sensitivity and specificity were also calculated in R 4.4.2.

#### Appendix A.1.3. Area Under the Curve (AUC) and Receiver Operating Characteristic Curve (ROC)

Results from Figure A6:

- From the plot, the closer the ROC curve is to the upper left corner, the better the model.
- The closer the AUC is to 1, the better the model.
- Based on the ROC curve and the AUC, the final model is good to very good, is appropriate for the data, and is useful to predict whether a child is seropositive.

#### Appendix A.1.4. Variance Inflation Factors (VIFs) to Assess Multicollinearity

**Table A8:** Variance inflation factors (VIFs) of variables in the final model.

Variables	VIF
Age of mother in years (centered)	1.14
Recruitment region	1.20
Household size	1.20
COVID-19-positive household contacts	1.14
Mother COVID-19 vaccination	1.56
Social activities of children during the COVID-19 pandemic	1.73
Use of mask by child in social activities	1.11
Distancing practice by child in social activities	1.37
COVID-19 diagnosis by healthcare provider	1.22

VIF, Variance inflation factor. No evidence of multicollinearity, VIFs < 5.

#### Appendix A.1.5. Hosmer and Lemeshow Goodness of Fit Test

H_0_: there is no lack of fit

H_A_: there is lack of fit

Chi^2^ test is not statistically significant (*p* = 0.87), indicating no evidence of lack of fit.
