# Supplementary material for: SARS-CoV-2 Seroprevalence of Surinamese Children and Determinants of Seropositivity in the CCREOH/MeKiTamara Cohort
Source: Children (Basel). 2026 Mar 31;13(4):493. doi: 10.3390/children13040493 (PMC13114446; doi:10.3390/children13040493)
Supplement: Supplementary file 1 [file children-13-00493-s001.zip › children-4171782-supplementary.pdf]

Caribbean Consortium for Research in Environmental and Occupational Health (CCREOH)

Impacts of COVID-19 on Surinamese children

Recruiter initials __________________

Instructions

This form has 4 sections:
- *Section A: Demographics*
- *Section B: COVID-19 Prevention*
- *Section C: COVID-19 Infection*
- *Section D: COVID-19 Vaccination*

Section A. Demographics

1. Study ID

____

2. Survey DOC (Date of Collection)

__ __ (dd)/ __ __ (mm)/ __ __ __ __ (yyyy)

3. Date of Birth mother

__ __ (dd)/ __ __ (mm)/ __ __ __ __ (yyyy)

4. Date of Birth child

__ __ (dd)/ __ __ (mm)/ __ __ __ __ (yyyy)

5. What is the gender of the child?

- ◯ 01 Female
- ◯ 02 Male

6. What is your ethnic group? ***(Mark all that apply)***

- ☐ 01 Chinese
- ☐ 02 Creole
- ☐ 03 Hindustani
- ☐ 04 Indigenous/Amerindian
- ☐ 05 Javanese
- ☐ 06 Caucasian
- ☐ 07 Maroon
- ☐ 08 Other, specify ____________

7. In which district do you live?

- ○ 01 Paramaribo
- ○ 02 Wanica
- ○ 03 Nickerie
- ○ 04 Saramacca
- ○ 05 Commewijne
- ○ 06 Para
- ○ 07 Coronie

8. In which resort do you live now?

- ○ 01 Beekhuizen
- ○ 02 Blauwgrond
- ○ 03 Centrum
- ○ 04 Flora
- ○ 05 Latour
- ○ 06 Livorno
- ○ 07 Munder
- ○ 08 Pontbuiten
- ○ 09 Rainville
- ○ 10 Tammenga
- ○ 11 Weg naar Zee

- 12 Welgelegen
- 13 De Nieuwe Grond
- 14 Domburg
- 15 Houttuin
- 16 Koewarasan
- 17 Kwatta
- 18 Lelydorp
- 19 Saramacca Polder
- 20 Groot Henar
- 21 Nieuw-Nickerie
- 22 Oostelijke Polders
- 23 Wageningen
- 24 Westelijke Polders
- 25 Brownsweg
- 26 Centrum
- 27 Klaaskreek
- 28 Kwakoegron
- 29 Marshallkreek
- 30 Sarakreek
- 31 Calcutta

- ○ 32 Groningen
- ○ 33 Jarikaba
- ○ 34 Kampong Baroe
- ○ 35 Tijgerkreek
- ○ 36 Wayamboweg
- ○ 37 Alkmaar
- ○ 38 Bakie
- ○ 39 Margaretha
- ○ 40 Meerzorg
- ○ 41 Nieuw-Amsterdam
- ○ 42 Tamanredjo
- ○ 43 Bigi Poika
- ○ 44 Carolina
- ○ 45 Noord
- ○ 46 Oost
- ○ 47 Zuid
- ○ 48 Johanna Maria
- ○ 49 Totness
- ○ 50 Welgelegen

9. How many people currently live in your home (including you)?

____

10. How long have you lived in your current home? (Please specify time in months)

____

11. Have you moved in the last 12 months?

○ 01 Yes ***(If yes, answer Section A, questions 12 and 13)***

○ 02 No

12. If yes, in which resort/village did you live before?

____

13. For how long did you live in your previous residence? (Please specify time in months)

____

14. What is your highest level of education completed?

○ 01 None

○ 02 Primary school

○ 03 Lower secondary school

○ 04 Technical vocational training

○ 05 Secondary school

○ 06 Higher education (Masters/Bachelor)

○ 07 Other, specify _____________

15. What is your employment status? ***(Mark all that apply)***

- 01 Employed for wages
- 02 Self-employed
- 03 Unemployed - looking for work
- 04 Unemployed - not looking for work
- 05 Homemaker/Stay at home parent
- 06 Student
- 07 Retired
- 08 Unable to work due to disability
- 09 Other

16. If working, do you:

- 01 Work in-person only
- 02 Work remotely only
- 03 Work both in-person and remotely

Instructions

*For all sections of this questionnaire, **"COVID-19 pandemic"** refers to the time period of February 2020 through February 2022.*

Section B. COVID-19 Prevention

1. How concerned were you about any of the following situations during the COVID-19 pandemic?

	Very concerned	Somewhat concerned	Not concerned	Not applicable
Contracting COVID-19 at work? (For example: hospital, office, and other work settings that are not your home)	○ 01	○ 02	○ 03	○ 04
Contracting COVID-19 outside of work? (For example: at the grocery store, when you are using transportation, or in other aspects of your daily life)	○ 01	○ 02	○ 03	○ 04
Infecting your family or friends with COVID-19	○ 01	○ 02	○ 03	○ 04

2. Compared to the time of the COVID-19 pandemic, how concerned are you now about the following situations?

	Less concerned	Same amount of concern	More concerned	Not applicable
Contracting COVID-19 at	○ 01	○ 02	○ 03	○ 04

work? (For example: hospital, office, and other work settings that are not your home)				
Contracting COVID-19 outside of work? (For example: at the grocery store, when you are using transportation, or in other aspects of your daily life)	○ 01	○ 02	○ 03	○ 04
Infecting your family or friends with COVID-19	○ 01	○ 02	○ 03	○ 04

3. How often did you obey curfew time when one was required during the COVID-19 pandemic?

Never	Rarely	Sometimes	Often	Always
○ 01	○ 02	○ 03	○ 04	○ 05

4. What did you do to protect you and/or your family from the coronavirus during the COVID-19 pandemic? ***(Mark all that apply)***

- ☐ 01 Wash your hands for 20 seconds with soap and water
- ☐ 02 Use sanitizers
- ☐ 03 Wear a mask to cover nose and chin when out of the house
- ☐ 04 Stand 6 feet apart from people
- ☐ 05 Stay at home (unless it was absolutely necessary to go out)
- ☐ 06 Not touch your face
- ☐ 07 Use home medicines to protect health
- ☐ 08 Change your diet
- ☐ 09 None of the above
- ☐ 10 Other, please specify ______________

5. What do you do now to protect you and/or your family from the coronavirus? ***(Mark all that apply)***

- ☐ 01 Wash your hands for 20 second with soap and water
- ☐ 02 Use sanitizers
- ☐ 03 Wear a mask to cover nose and chin when out of the house
- ☐ 04 Stand 6 feet apart from people
- ☐ 05 Stay at home (unless it is absolutely necessary to go out)
- ☐ 06 Not touch your face
- ☐ 07 Use home medicines to protect health
- ☐ 08 Change your diet
- ☐ 09 None of the above
- ☐ 10 Other, please specify ______________

The following questions are about disruptions to the child's social life and related social distancing practices resulting from the COVID-19 pandemic. These questions refer to the time period of February 2020 through February 2022. All questions are about your child enrolled in the CCREOH cohort.

6. Which of the following did the child do during the COVID-19 pandemic? (***Mark all that apply***)

- ☐ 01 Went out to restaurants for eat-in service
- ☐ 02 Went to essential stores (e.g., grocery store, pharmacy)
- ☐ 03 Went to non-essential stores (e.g., retail, clothing store)
- ☐ 04 Attended in-person religious services (church, mosque, synagogue, mandir)
- ☐ 05 Attended in-person community events
- ☐ 06 Attended/participated in in-person sports-related activities
- ☐ 07 Went to outdoor public places (e.g., playground, park, beach, zoo)
- ☐ 08 Went to indoor public places (e.g., library, movie theater)
- ☐ 09 Attended in-person social gatherings (e.g., birthday parties, playdates)
- ☐ 10 Went to a friend's house/invited a friend over
- ☐ 11 Went on an airplane
- ☐ 12 Traveled to a different district or country
- ☐ 13 The child did not do any of these

6.a. When the child did any of these activities, how often did your child do the following <u>during the COVID-19 pandemic</u>?

	Never	Rarely	Sometimes	Often	Always
Wear a mask to cover nose and chin	○ 01	○ 02	○ 03	○ 04	○ 05
Stand 6 feet apart from people	○ 01	○ 02	○ 03	○ 04	○ 05

Section C. COVID-19 Infection

For the following questions, "healthcare provider" means a doctor, nurse practitioner, physician assistant or anyone you go to for medical care. All questions are about your child enrolled in the CCREOH cohort, unless otherwise specified.

1. Has a healthcare provider ever told you that your child had, or likely had, COVID-19 (Coronavirus)?

○ 01 Yes

○ 02 No

2. Has your child ever tested positive for COVID-19?

○ 01 Yes

○ 02 No

3. Has your child had the nose swab test for the virus that causes COVID-19? ***(Mark all that apply)***

- ☐ 01 No, I never tried to get the child tested
- ☐ 02 No, I tried to get the child tested but was not able to
- ☐ 03 Yes, and the test showed that the child does not have it (**"negative"** test)

If yes → **3.a.** When was the date of the child's most recent **negative** test __ __ (mm)/__ __ __ __ (yyyy)

- ☐ 04 Yes, and the test showed that the child does have it (**"positive"** test)

If yes → **3.b.** When was the date of the child's most recent **positive** test __ __ (mm)/__ __ __ __ (yyyy)

3.c. Which nose swab test did your child have? ***(Mark all that apply)***

- ☐ 01 PCR test
- ☐ 02 Antigen test
- ☐ 03 I am not sure

4. Did your healthcare provider advise you to get your child's blood tested for COVID-19?

○ 01 Yes ***(If yes, answer Section C, question 5)***

○ 02 No

5. Has your child had a blood test to see whether he/she already had the COVID-19 virus ("serology")? ***(Mark all that apply)***

☐ 01 No, I never tried to get the child tested

☐ 02 No, I tried to get the child tested but was not able to

☐ 03 Yes, and the child is waiting for the results

If yes → **5.a.** When was the date of the child's most recent test __ __ (mm)/__ __ __ __ (yyyy)

☐ 04 Yes, and the test showed that the child did not have it (**"negative"** test)

If yes → **5.b.** When was the date of the child's most recent **negative** test __ __ (mm)/__ __ __ __ (yyyy)

☐ 05 Yes, and the test showed that the child did have it (**"positive"** test)

If yes → **5.c.** When was the date of the child's most recent **positive** test __ __ (mm)/__ __ __ __ (yyyy)

6. Which of the following symptoms did your child have when they were most recently tested for COVID-19 virus? ***(Mark all that apply)***

☐ 01 Fever or chills

☐ 02 Cough

☐ 03 Shortness of breath

- ☐ 04 Sore throat
- ☐ 05 Headache
- ☐ 06 Muscle or body aches
- ☐ 07 Runny nose
- ☐ 08 Fatigue or excessive sleepiness
- ☐ 09 Diarrhea, nausea, or vomiting
- ☐ 10 Loss of sense of smell or taste
- ☐ 11 Itchy/red eyes
- ☐ 12 None of the above ***(If "None of the above", answer Section C, question 7 and 8)***

6.a. Which of the following occurred as a result of your child's symptoms? ***(Mark all that apply)***

- ☐ 01 The child was kept overnight in a hospital because a healthcare provider thought he/she had COVID-19
- ☐ 02 The child saw a healthcare provider in person, such as in a clinic, doctor's office, urgent care, or Emergency Room (ER)/Emergency Department (ED)
- ☐ 03 You/the child spoke to a healthcare provider over the phone, by email, or online
- ☐ 04 You/the child self-isolated or quarantined at home
- ☐ 05 None of the above

6.b. In the two weeks before your child’s symptoms, did he/she: ***(Mark all that apply)***

- ☐ 01 Have contact with someone who tested positive for COVID-19
- ☐ 02 Have contact with someone who likely had COVID-19 (e.g., was not tested but had symptoms; was told by a healthcare provider that he/she likely had it)
- ☐ 03 Travel to a different district or country (please specify: _____________)
- ☐ 04 None of the above
- ☐ 05 I don’t remember

7. In what ways has the COVID-19 pandemic affected your child’s overall healthcare/medical care? ***(Mark all that apply)***

- ☐ 01 The child did not go to healthcare/medical care appointments because I was concerned about the child entering the healthcare provider’s office
- ☐ 02 The child’s healthcare provider canceled appointments
- ☐ 03 The child’s healthcare provider changed to phone or online visits
- ☐ 04 The child’s healthcare provider told him/her to self-isolate or quarantine
- ☐ 05 None of the above

The following questions are about your experience with COVID-19, or the coronavirus. For each question, do the best you can to remember the details requested.

8. Have you or anyone else living in your home tested positive for COVID-19 at any point in time?

◯ 01 Yes ***(If yes, answer question 9)***

◯ 02 No

9. If "yes", describe the level of care you or those living in your home received:

◯ 01 Did not seek medical care

◯ 02 Received medical care but was not hospitalized

◯ 03 Was hospitalized or admitted to a healthcare facility

Section D. COVID-19 Vaccination

1. Currently, COVID-19 vaccines for children under the age of 12 years old are not authorized in Suriname. If the COVID-19 vaccine were available for children under 12 years old, would you vaccinate your child?

- ○ 01 Yes
- ○ 02 No
- ○ 03 Not sure

2. If “yes”, why? ***(Mark all that apply)***

- ☐ 01 Risk of infection with COVID-19
- ☐ 02 COVID-19 is a serious disease
- ☐ 03 Protect the people around
- ☐ 04 COVID-19 vaccine is effective
- ☐ 05 Government publicity
- ☐ 06 Doctor's recommendation
- ☐ 07 Others

3. If “no/not sure”, why? ***(Mark all that apply)***

- ☐ 01 Concern about vaccine’s safety or side effects
- ☐ 02 No risk of infection with COVID-19
- ☐ 03 Distrust vaccination clinics, hospitals, or other medical offices
- ☐ 04 Distrust vaccine manufacturers
- ☐ 05 Poor service quality of healthcare system
- ☐ 06 Generally reluctant to vaccinate
- ☐ 07 No professional gave me a detailed introduction to the vaccine
- ☐ 08 Conflict with my personal beliefs
- ☐ 09 I live far from a vaccination clinic
- ☐ 10 I (or someone I know) had adverse experience with the vaccine
- ☐ 11 Want to immunize in a natural way rather than using a vaccine
- ☐ 12 Doubt the vaccine’s effectiveness
- ☐ 13 I get negative information about the vaccine in the media
- ☐ 14 I don’t have time
- ☐ 15 Vaccination contraindications (example: a severe allergic reaction to vaccines)
- ☐ 16 Others

4. Have you received the COVID-19 vaccine?

- ○ 01 Yes
- ○ 02 No
- ○ 03 Not sure
- ○ 04 Prefer not to answer

5. If “yes”, why? ***(Mark all that apply)***

- ☐ 01 Risk of infection with COVID-19
- ☐ 02 COVID-19 is a serious disease
- ☐ 03 Protect the people around me
- ☐ 04 COVID-19 vaccine is effective
- ☐ 05 Government recommendation
- ☐ 06 Doctor's recommendation
- ☐ 07 Others

6. If “no/not sure/Prefer not to answer”, why? ***(Mark all that apply)***

- ☐ 01 Concern about vaccine’s safety or side effects
- ☐ 02 No risk of infection with COVID-19
- ☐ 03 Distrust vaccination clinics, hospitals, or other medical offices

- ☐ 04 Distrust vaccine manufacturers
- ☐ 05 Poor service quality of healthcare system
- ☐ 06 Generally reluctant to vaccinate
- ☐ 07 No professional gave me a detailed introduction to the vaccine
- ☐ 08 Conflict with my personal beliefs
- ☐ 09 Cannot acquire the vaccine
- ☐ 10 I live far from a vaccination clinic
- ☐ 11 I (or someone I know) had adverse experience with the vaccine
- ☐ 12 Want to immunize in a natural way rather than using a vaccine
- ☐ 13 Doubt the vaccine's effectiveness
- ☐ 14 I get negative information about the vaccine in the media
- ☐ 15 I don't have time
- ☐ 16 Vaccination contraindications (example: a severe allergic reaction to vaccines)
- ☐ 17 Others
- ☐ 18 Prefer not to answer

Comments: ___________________________

Were blood spots collected from the child today?

○ 01 Yes

○ 02 No

If not, what is the reason _________

Thank you for your cooperation!

End of survey
